# Manual Therapy and Exercise for the Management of Chronic Neck Pain With Multiple Neurovascular Comorbidities: A Case Report

**DOI:** 10.7759/cureus.36961

**Published:** 2023-03-31

**Authors:** Zach Mayo, Christopher Keating

**Affiliations:** 1 Orthopaedics, The Training Room, Cherry Hill, USA; 2 Orthopaedics, Thomas Jefferson University, Philadelphia, USA

**Keywords:** chiari malformations, postural tachycardia syndrome, exercise training, physical therapy rehabilitation, craniocervical fusion, manual therapist, resistant migraine, chronic non-specific neck pain

## Abstract

This case describes the clinical decision-making behind the conservative clinical management of an individual presenting with chronic neck pain with myriad neuromuscular comorbidities. The focus of this case report is to support the safe utilization of manual therapy and describe the tolerable prescription of strength and endurance exercise in a patient with numerous complications to improve self-efficacy. A 22-year-old female college student presented with a chief complaint of chronic, non-specific neck pain with comorbid Chiari malformation, migraines, upper cervical spinal fusion, Ehlers-Danlos syndrome (EDS), and postural orthostatic tachycardia syndrome (POTS) to an outpatient physical therapy clinic for evaluation and treatment. Following four sessions of physical therapy treatment, no clinically significant improvement in the individual's symptoms and daily function was achieved. Despite the lack of measurable change, the patient reported the program's value on her ability to self-manage her complex condition. The patient responded well to manual therapy, specifically thrust manipulations. In addition, both endurance and strengthening exercises were well tolerated and provided a measure of self-management that may not have been achieved before physical therapy management. This case report highlights the need for exercise and pain-modulating interventions in highly complex individuals to reduce medical intervention by advancing the patient's self-efficacy. There is a need for further research about the utility of standardized outcome measures, joint manipulations, and the addition of cervico-ocular exercises for those who present with neck pain and pertinent neuromuscular comorbidities.

## Introduction

Neck pain is among the most prevalent conditions treated in outpatient physical therapy, accounting for an estimated 25% of all patient's cases [1]. Approximately 22-70% of the population will experience neck pain at some point in their lifetime, more commonly affecting women with the greatest prevalence in the fifth decade of life [1]. In comparison to acute complaints of neck pain, chronic cases are less common, with an estimated 12-month prevalence ranging from 2-11%. Chronic neck pain cases often result in prolonged disability and economic burden, making managing these conditions a vital area of research [2]. 

Various musculoskeletal, neuromuscular, and neurovascular conditions contribute to an individual's neck pain, which can significantly increase the complexity of clinical management, impact prognosis, and contribute to the condition's chronicity [1,2]. 

One example of a condition that may contribute to neck pain is a migraine, a debilitating type of primary headache [3]. An estimated 70% of individuals who have migraines report concurrent neck pain [4]. Research indicates the relationship between cervical pain and migraines may be due to the sensitization of the trigeminal-cervical nucleus, which receives converging, nociceptive input from the occipital nerve roots and the ophthalmic branch of the trigeminal nerve [5]. Despite possible musculoskeletal contributions, the primary mechanism of migraine-related pain is thought to be neurovascular via the trigeminovascular system [6]. Therefore, treating migraine-related neck pain through a solely musculoskeletal approach may be insufficient to address the complex neurophysiological cascade that likely underlies it. 

Type I Chiari malformation, a developmental condition that causes excessive compression forces and partial displacement of the cerebellum into the spinal canal, is one other condition that may present with neck pain. Approximately 80% of individuals with a Type I Chiari malformation have a chief complaint of neck pain, with other symptoms including cerebellar dysfunction and ocular and otoneurologic disturbances [7]. Suboccipital decompression surgery is the most common surgical procedure for Type I Chiari malformation and is often accompanied by a C1-C2 cervical fusion due to inherent bony abnormality [8]. An upper cervical fusion can continue predisposing an individual to neck pain despite addressing the neurological symptoms. It permanently limits motion at the upper cervical spine, often leading to excessive cervical segmental mobility and loading throughout the lower cervical spine. 

Ehlers-Danlos syndrome (EDS) is a collection of congenital connective tissue disorders characterized by hypermobile joints and soft elastic tissue and is commonly seen in individuals with Chiari malformation. Individuals with EDS commonly present with muscular weakness and diminished proprioception and the hypermobility subtype of EDS has recently been associated with early chronic pain onset and hyperalgesia [9,10]. Postural orthostatic tachycardia syndrome (POTS) is a common comorbid condition seen amongst those with EDS, migraines, or Chiari malformation; however, the mechanistic relationship between POTS and the pathologies mentioned above remains unclear [11]. 

The clinical management of an individual with chronic neck pain with migraines, EDS, Chiari malformation, POTS, and upper cervical spinal fusion presented a unique challenge. This case report will discuss an episode of care reflecting the clinical decision-making and limitations of current evidence and clinical tools. 

## Case presentation

A 22-year-old caucasian female college student presented with a chief complaint of chronic, non-specific neck pain with comorbid Type I Chiari malformation, migraines, upper cervical spinal fusion, EDS, fibromyalgia, scoliosis, and POTS to an outpatient physical therapy clinic for evaluation and treatment. She had a posterior fossa decompression with a C1-C2 fusion using a posterior approach 5 years before the consultation for physical therapy. The fusion was performed out of concern for upper cervical instability following the decompression procedure, provided her history of EDS. No other medical history was significant, and she is taking Bystolic (beta blocker) and Nurtec (migraine) medications. The Neck Disability Index (NDI) was utilized as the primary functional outcome measure and, at initial evaluation, reported a score of 29/50 [12]. She is a school teacher home from an international assignment for two weeks and wishes to have therapy to help her self-manage her condition abroad. 

Upon clinical examination, she reported 5/10 pain on average, 2/10 at best, and 9/10 at worst on the numeric pain rating scale. The dermatome, myotome, and deep tendon reflex testing results were normal, with a blood pressure of 110/78 mmHg and a heart rate of 60 bpm. The patient presented with reduced cervical mobility in multiple planes, discomfort at end-range bilaterally with thoracic rotation, craniocervical flexion test (CCFT), and deep neck flexor endurance test impairments (Table 1). Posteroanterior intervertebral mobility testing of the cervical spine included central and unilateral posteroanterior spring testing, and lateral glides were performed. The patient presented with pain and hypomobility with left posteroanterior spring testing at C5 and hypomobility at C3-C5 with lateral glides. Her history of a Type I Chiari malformation and EDS, combined with her reports of difficulty reading and concentrating for extended periods, warranted additional vestibular-ocular and proprioceptive testing (Table 1).

**Table 1:** Initial Examination Significant Findings

	Passive Range of Motion	Active Range of Motion	Strength	Endurance	Proprioception	Coordination
Cervical Flexion	Normal	Normal	5/5	Deep Neck Flexion Endurance Test: 28 seconds (Positive)	Craniocervical Flexion Test: 26 mmHg for 8 seconds (Positive)	Ocular Smooth Pursuit: Positive for the reproduction of symptoms
Cervical Left Rotation	0-60˚	0-60˚				
Cervical Right Rotation	0-60˚	0-45˚				
Cervical Thoracic	Rotation-Lateral Flexion Test: Negative	Normal with End Range Pain	3+/5 (middle and lower trapezius)	Unable to tolerate	Scapular Joint Position Error: Negative	

**Figure 1:**
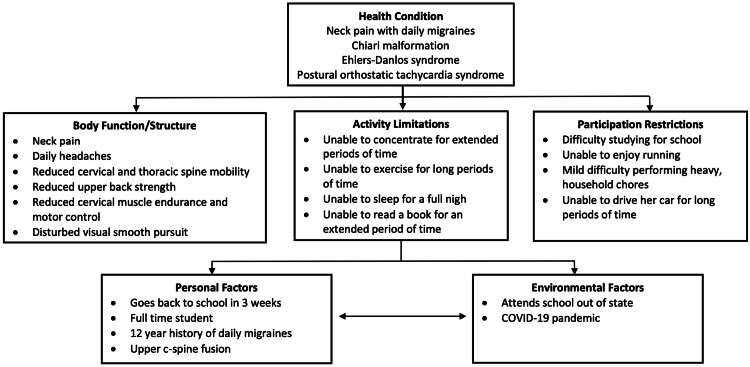
International Classification of Functioning, Disability and Health

By the clinical practice guidelines published by the orthopedic section of the APTA for neck pain, the patient fits the classification of chronic neck pain with mobility deficits [1]. It is common to see motor control and endurance deficits in those with chronic neck pain and mobility deficits. In addition to her neck pain, the patient's headache presentation was thought to be consistent with migraines without aura based on the International Headache Society criteria [13]. Her prognosis was deemed fair based on the chronicity of her symptoms, comorbidities, and the limited period for her episode of care due to personal factors (Figure 1).

**Figure 2:**
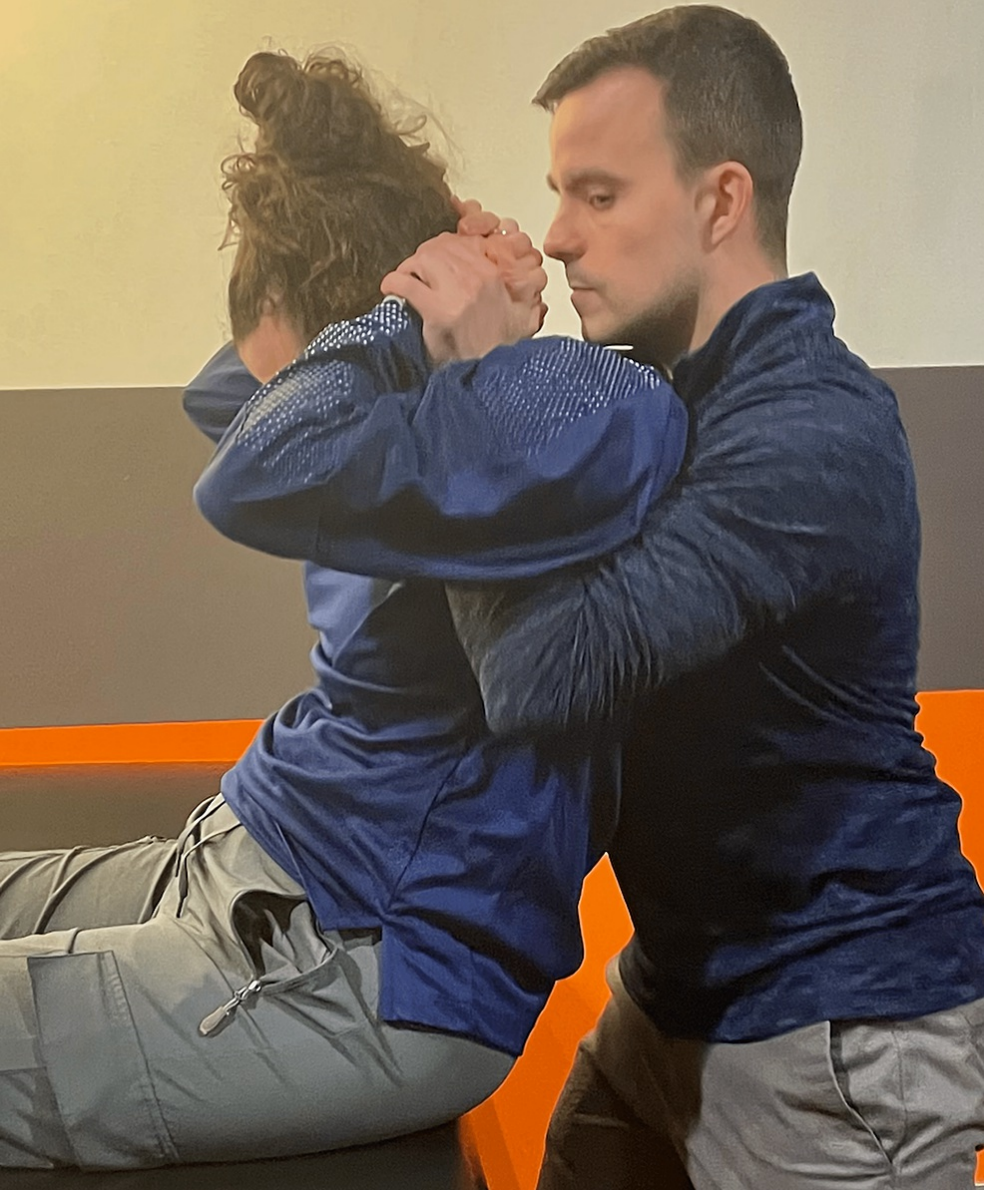
Cervicothoracic Thrust Manipulation

The patient's plan of care utilized a multi-modal approach, including manual therapy to the spine (cervical, cervicothoracic (Figure 2), and thoracic), neuromuscular re-education, endurance/strengthening of the cervicoscapulothoracic musculature, mobility exercises, and patient education (Table 2). Thrust and non-thrust spinal manipulation is a safe and effective component of a multi-modal approach to treating neck pain [14]. Joint manipulation is shown to have wide-ranging neurophysiological effects through supraspinal, spinal, and peripheral mechanisms leading to decreased pain levels and improved muscle activation [15]. Thrust manipulation to adjacent spinal segments has been safely performed in those with single and multilevel spinal fusion [16,17]. Evidence regarding the safety and efficacy of joint manipulation for those with EDS is scarce; however, case reports have demonstrated the safe application of low-amplitude joint manipulation as part of a multi-modal plan of care with no reported adverse effects in individuals with EDS [18]. 

Proprioceptive neuromuscular facilitation techniques addressed muscular contributions to the patient's reduced cervical mobility, as suggested by a significant difference in passive and active ROM. In all sessions, manual therapy was followed with cervicothoracic mobility and neuromuscular activation, endurance, and strengthening exercises targeting the scapular stabilizers, thoracic extensors, and deep neck flexors (Table 2). 

**Table 2:** Plan of Care and Interventions

Initial Evaluation	Sessions 2-3	Session 4	Discharge Instructions
Seated mid-thoracic thrust manipulation with flexion bias	Cervical anterior mobilizations (Grades II-III)	Cervical anterior mobilizations (Grades II-III)	Lateral flexion towel mobilization with movement
Seated cervicothoracic distraction thrust manipulation	1st rib mobilization (Grade III-IV)	1st rib mobilization (Grade III-IV)	Proprioceptive neuromuscular facilitation contract-relax stretch (5x5 seconds) for scalene and levator scapulae
Manual scalene Proprioceptive neuromuscular facilitation contract-relax stretch (5x 5 seconds) bilaterally	Upper thoracic and rib mobilization (Grade III-IV)	Upper thoracic and rib mobilization (Grade III-IV)	Seated wall angels (3x12-15)
Lateral flexion towel mobilization with movement (20 repetitions bilaterally)	Supine sub-occipital release (3x30 seconds)	Supine sub-occipital release (3x30 seconds)	Supine Deep Neck Flexor Endurance (10x10 seconds)
Side-lying thoracic rotations (20 reps bilaterally)	Side-lying thoracic rotations (20 repetitions bilaterally)	Side-lying thoracic rotations (20 repetitions bilaterally)	Continue physical therapy upon return to university
Scapular wall slides with lift-off (3x12-15 repetitions)	Bilateral shoulder resisted external rotation (3x12-15)	Bilateral shoulder resisted external rotation (3x12-15)	
	Alternating cable single arm rows (3x12)	Alternating cable single arm rows (3x12)	
	Wall angels (2x15)	Wall angels (2x15)	
	Supine Deep Neck Flexor Endurance (10x10 seconds)	Supine Deep Neck Flexor Endurance (10x10 seconds)	

The dosage of the selected exercises was based on the patient's experience with resistance training, a restricted period for her episode of care, chronicity, and EDS. We did not expect to achieve changes in muscular strength through structural adaptations. The exercise dosage was aimed at improving the neuromuscular component of strength. Neural adaptations that can contribute to improvements in strength can be achieved through various mechanisms; however, the patient's lack of previous experience with resistance training warranted careful consideration of her exercise programming. A prescription targeting higher volumes of targeted movement was selected to induce early neural adaptations while monitoring for adverse training effects and symptom exacerbation. 

The patient was seen for four sessions over two weeks, after which she returned to school. We observed no significant difference in primary (NDI 29% to 32%) or secondary outcome measures, as expected for short-term outcomes. At the end of her fourth visit, she was advised to continue physical therapy treatment upon returning to school. She was provided with a home exercise program to facilitate improved cervicothoracic mobility, motor control, and endurance. There were no adverse events in using thrust or non-thrust manipulations in managing this patient's condition during or after treatment. 

Long-term outcomes were collected through email exchanges to assess the physical therapy plan's impact on the patient. In the words of the individual described in this case report six months after discharge: 

"I have dealt with chronic daily headaches, migraines, neck pain, Chiari Malformation, EDS, POTS, and anemia for a decade. In late 2016, I had Chiari Malformation decompression and upper cervical fusion surgery. Since then, my neck, spine, and shoulder pain have increased. Regarding physical therapy, I have been to three clinics to reduce this pain. Although helpful, my pain has never been fully alleviated. Because of my chronic and overlapping conditions, I am fairly certain neck and back pain will always be a part of my everyday life. The stretches and exercises I have received through physical therapy combined with massages, yoga, topical treatments, and other medication all work to keep my pain manageable".

## Discussion

This case report demonstrates the safe utilization of different thrust and non-thrust manipulations on a patient with several complex neuromuscular conditions and facilitates increased self-efficacy through exercise. Following a two-week episode of care, the patient demonstrated no clinically significant improvement in her chronic neck pain using a multi-modal approach composed of manual therapy, cervicothoracic mobility exercises, cervicothoracic neuromuscular re-education, and endurance training. Time was a limiting factor in our ability to achieve significant changes in objective measures; therefore, we focused on improving self-efficacy and managing her complex condition. 

This report outlines the challenges to patient care for an individual with non-specific neck pain complicated by musculoskeletal and neurovascular comorbidities. One challenge area is the paucity of research on the conservative treatment of individuals with neck pain with impactful muscular and neuromuscular comorbidities. Furthermore, the psychometric properties of the clinical evaluation tools commonly used for individuals with neck pain are unknown for an individual with an upper cervical fusion and various comorbidities that may contribute to neck pain [19]. Therefore, the results of the objective measures used in this case report, such as the CCFT, deep neck flexor endurance test, and NDI, must be interpreted with caution, as they may not accurately assess their respective impairment and functional limitation domains. Further research is needed to discern the clinical utility of tests and measures for sub-populations of individuals with musculoskeletal and neuromuscular comorbidities. 

There is limited evidence on the safety and efficacy of manual therapy in individuals with EDS and cervical fusion. Only a handful of published case reports have used thrust manipulation in a multi-modal approach to care for those with EDS or cervical fusion [16]. The efficacy and safety of joint thrust manipulation on the EDS population, with or without cervical fusion, remains unknown and is reliant upon sound clinical decision-making and risk-benefit analysis from the clinician [9]. Despite a minimal short-term effect on her symptoms, this case report provides an isolated instance in which joint manipulation was safely performed on an individual with EDS. 

Cervical spine manipulation has been an area of research interest for the conservative treatment of migraines due to its established benefits for cervicogenic headaches through its proposed neurophysiological impact on the afferent C1-C2 nerve fibers and processing at the trigeminocervical nucleus. Rist and colleagues concluded that cervical manipulation might be an effective therapeutic intervention to reduce pain intensity and the number of symptomatic days in those with migraines [20]. Overall, additional research on the efficacy and safety of joint manipulation in the EDS population and those with migraines is needed to guide clinical practice. 

The revised clinical practice guidelines for neck pain published by the APTA were utilized to direct treatment in this patient's case; however, its use and treatment recommendations provided limited efficacy for a case involving multiple neurovascular and musculoskeletal comorbidities [1]. The established guidelines for treatment are designed for those with neck pain in the absence of other impactful conditions such as migraines and an upper cervical spinal fusion. Therefore, while it helped to guide intervention from an orthopedic standpoint, the current guidelines were not suited to guide the entirety of the clinical decision-making performed during this case, as many of the clinical assessment tools, intervention strategies, and diagnostic recommendations are inapplicable and lack sufficient evidence for the constellation of chronic conditions encountered in this case.

## Conclusions

There is limited research to guide the management of highly complex patients presenting with neurovascular and musculoskeletal conditions. In this case, evidence-based practice highlights the need for clinicians to utilize a patient-centered approach where clinical decisions are based on the best available evidence, clinician experience, and patient beliefs and values. Through this approach, the clinician was able to have a profound impact on how the patient can address their complex condition in a safe and empowering manner. While objective tests and measures of the case report did not find a statistically significant difference in short-term assessment, the long-term qualitative report of the patient provided salient support for the interventions provided. The utilization of thrust manipulation is not endorsed for all patients with EDS or cervical fusion but for special cases when the proper progression of force and clinical reasoning is applied with the patient's informed consent of the risks and benefits.
